# Bacteriophage-Delivering Hydrogels: Current Progress in Combating Antibiotic Resistant Bacterial Infection

**DOI:** 10.3390/antibiotics10020130

**Published:** 2021-01-29

**Authors:** Hyun Young Kim, Rachel Yoon Kyung Chang, Sandra Morales, Hak-Kim Chan

**Affiliations:** 1 Advanced Drug Delivery Group, Faculty of Medicine and Health, School of Pharmacy, The University of Sydney, Sydney, NSW 2006, Australia; hkim3443@uni.sydney.edu.au (H.Y.K.); yoon.chang@sydney.edu.au (R.Y.K.C.); 2 Phage Consulting, Sydney, NSW 2006, Australia; morales.sandra@gmail.com

**Keywords:** bacteriophage (phage), hydrogel, multidrug-resistant bacteria, formulation, efficacy, safety, stability

## Abstract

Antibiotic resistance remains as an unresolved global challenge in the health care system, posing serious threats to global health. As an alternative to antibiotics, bacteriophage (phage) therapy is rising as a key to combating antibiotic-resistant bacterial infections. In order to deliver a phage to the site of infection, hydrogels have been formulated to incorporate phages, owing to its favorable characteristics in delivering biological molecules. This paper reviews the formulation of phage-delivering hydrogels for orthopedic implant-associated bone infection, catheter-associated urinary tract infection and trauma-associated wound infection, with a focus on the preparation methods, stability, efficacy and safety of hydrogels as phage carriers.

## 1. Introduction

Bacteria are developing resistance against commercial antibiotics at an alarming rate, and antibiotic resistance is now one of the biggest threats to global health, contributing to rise in morbidity and mortality [1,2]. Indiscriminate use of antibiotics has led to the emergence of multidrug-resistant (MDR) bacteria with increased pathogenicity [3]. Thus, novel therapeutics are urgently needed to address the consequence of MDR infections. Bacteriophage (phage) therapy is being reconsidered as a potential alternative or adjunctive therapy to conventional antibiotics due to its ability to treat infections associated with MDR bacteria [4]. Phages are viruses that specifically target bacteria without hindering commensal microbiome [1,5]. Obligatory lytic (virulent) phages are utilized in phage therapy. Lytic phages inject their genetic material into the bacteria upon receptor recognition, self-replicate and then burst release their progenies during bacteriolysis [1,6]. Unlike some antibiotics, which are known to elicit more severe side effects in the patient than the infection itself [7,8], phage therapy is considered generally safe with no severe side effects reported in humans [1]. Other advantages include the ability to co-administer phages with antibiotics to induce synergistic antimicrobial effect [9,10,11] and to penetrate bacterial biofilms both in vitro and in vivo [12,13,14], further expanding the role of phage therapy. Biofilm formation on medical devices has posed significant problems to healthcare systems not only due to the emergence of MDR pathogens but also the antibacterial shielding effect of extracellular polymeric substances [15]. Phages produce enzymes which degrade extracellular polymeric substances, thereby being able to target persistent bacteria that are difficult to kill with antibiotics [16]. Therefore, phage therapy holds promising potential to help ease the burden of MDR bacterial infections. Considering its favorable antibacterial effects, phage therapy has been developed and approved as a standard medical application in Russia, the Republic of Georgia and other Former Soviet Union countries for many decades [17]. Moreover, phage therapy has undergone at least four Phase 1 and 2 clinical trials in the last 10 years to further expand its application to reach the market in the West [6]. Clinical case studies have investigated direct application in liquid formulation of phages to sinuses, wound and ear infection sites [18,19,20,21]. Although promising, liquid formulations lacking controlled delivery of phages can be therapeutically limited by the sudden release and rapid dispersion and/or elimination of phages from the desired microenvironment.

Formulation of phages involve a dual challenge of ensuring phage biostability and physical stability of the formulation (solution, suspension, gel or powder). Phages are, in a sense, large protein complexes enclosing genetic materials (DNA or RNA) and are only partially stable in solution, like most proteins. Naturally, protein stabilization strategies need to be considered, and they have in fact been applied in formulating phage therapeutics [22]. To become a viable therapeutic product, both native structure and biological activity of phages in the formulations must be retained during production and storage. Individual phages may have different stability profiles even in the same formulation [23,24,25], adding further complexity. To date, the development of stable phage formulations for therapeutic purposes is still an underexplored area of research. Hydrogels have been used as a vehicle to enable controlled delivery or administration of biologics such as phages to the target site of interest, including wounds [26,27,28] and implants [29,30,31]. Hydrogels are non-toxic polymeric materials exhibiting three-dimensional networks along with their hydrophilic characteristics playing an essential role in containing large water content, which serves as a biocompatible environment suited for biological molecules [32,33]. Moreover, hydrogels resemble living tissues by holding a high proportion of water content within its matrix, providing optimal environment for accommodating proteins, living cells and other biomolecules, hence expanding its application in biomedical field [33,34]. In addition, hydrogel system enables controlled release of drugs through their tunable physical properties and biodegradability [35], which is also applicable to biomolecules delivery [36]. Considering the favorable characteristics for incorporating biological agents, hydrogels are a promising vehicle for the delivery of phages.

Exploiting the benefits of both phages and hydrogels, phage hydrogels have been utilized to treat and/or prevent MDR bacterial infections. A growing number of in vitro and in vivo preclinical studies indicate that hydrogels could be an ideal phage delivery system. This review focuses on the formulation of phages as an active ingredient in hydrogel formulations. We will discuss recent progress in the formulation of hydrogels as a phage carrier and cover the production methods of phage hydrogels, followed by the stability of phages in hydrogels. Finally, we will discuss the efficacy and safety of phage hydrogels both in vitro and in vivo.

## 2. Production Methods of Phage-Delivering Hydrogels

### 2.1. Alginate Hydrogel

Alginate is a natural polymer that can form hydrogels with good biodegradability and biocompatibility, low toxicity and ease of gelation (Table 1 [37]. It is widely used in commercial wound dressing, as it facilitates wound healing while minimizing bacterial infections. Beyond topical application, alginate hydrogels can be injected into the body with minimal invasiveness [37]. Injectable alginate hydrogels have been reported as a strategy to locally deliver phages for preventing and/or treating orthopedic implant-associated infections [29,31].

**Figure 1:**
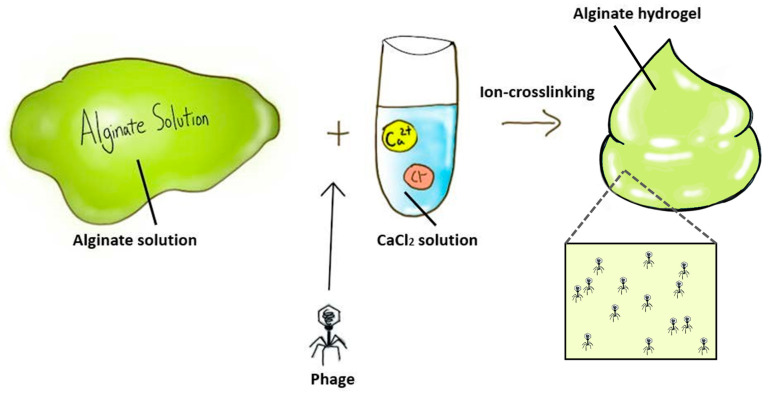
Preparation of phage-delivering alginate hydrogel formulation.

The most common method of formulating alginate hydrogels is adding ionic cross-linking agents such as divalent cations into an aqueous alginate solution (Alg) [37]. For example, Cobb et al. mixed filter-sterilized 3% (*w*/*v*) Alg with genetically modified phages (e.g., CRISPR/Cas9 system integration, virulence genes removal and host specificity expansion), followed by crosslinking with 0.21% (*w*/*v*) CaSO_4_ solution to produce 2% (*w*/*v*) alginate hydrogel containing phage at 3 × 10^7^ plaque-forming units (PFU)/mL [31]. Similarly, Barros et al. suspended phages vB_EfaS_LM99 (LM99) in 2% (*w*/*v*) Alg, and then the mixture was dropped into 250 mM CaCl_2_ solution to achieve a phage hydrogel at 1.0 × 10^7^ PFU/mL (Figure 1) [29]. Although divalent ions such as Ca^2+^ and Mg^2+^ are known to promote phage stability [49,50], the addition of a crosslinker such as CaCl_2_ can also cause a significant drop in the phage titer (>1 log) [29]. This appears to be phage-dependent and could be due to the depletion of calcium ions for phage interaction and/or the crosslinking step itself and the associated chemical and mechanical changes of the hydrogels. Cumulative release of phage LM99 was dependent on the pH of alginate hydrogels, which was proportional to the swelling ability of the polymer network. At pH 3, the hydrogel matrix shrunk, and no phage release was observed. However, the absence of viable phages released in hydrogels with a low pH could be due to phage inactivation rather than poor release, or a combination of both. At physiological pH, 40% of phages were released after 30 min, followed by 97% release at 24 h.

In both studies above, phages were mixed with Alg prior to adding the crosslinking agent. The effects of crosslinking agents and the crosslinking process have been poorly studied. Perhaps incorporating phages into formulated alginate hydrogels could avoid potential adverse interactions between the crosslinking agents and the target phages. While studies have focused on alginate hydrogel as injectables, the formulation could readily be developed as a vehicle for topical application of phages.

### 2.2. PEG (Polyethylene glycol) Hydrogels

Polyethylene glycol (PEG) hydrogels are one of the most commonly used synthetic hydrogels in the biomedical field, owing to their adjustable physicochemical properties, minimal protein adsorption and low toxicity (Table 1) [51]. PEG hydrogels can be combined with different functional groups and crosslinking moieties to produce various formulations.

#### 2.2.1. PEG-4-MAL (Polyethylene Glycol-4-Maleimide) Hydrogel

Poly(ethylene glycol)-4-maleimide (PEG-4-MAL) hydrogel is driven from chemically functionalized PEG hydrogel via Michael-type addition where free radical initiators are not required, as compared with other hydrogels such as hyaluronic acid methacrylate hydrogel which require photo-initiator for crosslinking (Table 1) [40,48]. Wroe et al. mixed an adhesive peptide and a crosslinker in buffer, and then, phages (1.2 × 10^8^ PFU/mL) active against *Pseudomonas* (ΦPaer4, ΦPaer14, ΦPaer22, ΦW2005A) or *Staphylococcus* (Φ4) were added. The resulting phage mixture was combined with 4.0% (*w*/*v*) PEG-4-MAL macromers (20-kDa) at pH 6.0–6.5 to produce a hydrogel [30]. Each ingredient possesses a unique function in PEG-4-MAL hydrogel formulation. In detail, cell adhesion peptides (short amino acid sequences) functionalize PEG-macromer to spread and attach to a 3D cell, enabling encapsulated biologics at the target site upon gel degradation [51]. Protease degradable peptide crosslinkers play an important role in tuning the biodegradability of hydrogel matrix [30], which ultimately controls the rate of phage release. Wroe et al. found that of the crosslinkers tested, protease-degradable peptide GCRDVPMSMRGGDRCG (VPM), which was used in the reaction, exhibited the highest rate of phage release [30]. The rate of phage release could be further fine-tuned by adjusting the ratio between VPM (degradable) and dithiothreitol (non-degradable), which controlled the degradation rate of hydrogels [52]. Controlling the rate of viable phage release is important for achieving a therapeutic dose in vivo. Maleimide functional group in PEG-4-MAL macromers provides rapid reaction kinetics and induce the shortest gelation time among other PEG hydrogels, which makes it suitable for clinically in situ gelation [51]. Furthermore, PEG-4-macromers have high specificity for thiols at physiological pH, which is ideal for Michael-type addition, creating bioactive hydrogel networks. Since PEG-4-MAL macromers form robust networks at low polymer weight percentages, this can reduce the stiffness of the hydrogel [51].

#### 2.2.2. PEG (Polyethylene Glycol)-Polyurethane Hydrogel

PEG hydrogel crosslinked with polyurethane matrix has been used to apply phages to catheters to prevent urinary tract infections [53,54,55,56]. Phages are anchored to the surface of the PEG hydrogel by forming a covalent bond via urethane linkage between the amine group on the surface of phages and hydroxyl group on PEG hydrogel [57]. Hence, phages can adsorb to PEG hydrogel-coated catheters and exert an antimicrobial effect. A polyurethane matrix is used as part of the catheter coating due to its hydrophilic, smooth and easily injectable properties [44]. PEG-polyurethane hydrogel possesses an anti-adhesive property [45], preventing biofilm formation on catheters. Both single phages and a cocktail of phages have been formulated with this type of hydrogel on catheters to target various pathogens, such as *Pseudomonas aeruginosa* [53], *Staphylococcus epidermidis* [55], *Escherichia coli* [54] and *Proteus mirabilis* [54]. The formulation process was quite simple, where the hydrogels on catheters were pretreated with phages (approximately 10^10^ PFU/mL) for 1–2 h at 37 °C [53,55], giving a mean concentration of 1.4 × 10^6^ PFU/cm^2^ [53]. On the other hand, Carson et al. treated the hydrogel-coated Foley catheter with phages at much lower titer (1.0 × 10^6^ PFU/mL) [54]. Although the final titer in the gel was unreported, it was expected to be around 100 PFU/cm^2^ based on other studies. Lehman and Donlan treated each catheter segment with an anti-*Pseudomonas* phage cocktail (1.0 × 10^9^ PFU/mL) and an anti-*Proteus* phage (3.0 × 10^8^ PFU/mL), followed by incubation for 1 h at 35 °C [56]. Although the phage titer used to pretreat the catheters was always reported, the final concentration of phages incorporated in the hydrogel and the release rate of these phages were almost never studied. Nonetheless, the ease of formulation development is attractive as they could be easily applied on site at hospitals.

### 2.3. PVA (Polyvinyl Alcohol)-Eudragit^®^ S 100 Hydrogel

Polyvinyl alcohol (PVA) is a synthetic polymer and its hydrogels are mainly prepared by physical crosslinking method (freeze-thaw method), as no by-products are produced in the process, minimizing the risk of toxicity from crosslink remnants (Table 1) [42]. Milo et al. mixed a *Proteus* phage (1.0 × 10^10^ PFU/mL) with hydrogel to produce a final PVA concentration of 10% [43]. The catheter was coated with phage hydrogel and stored at −20 °C overnight for crosslinking, then coated with pH-sensitive Eudragit^®^ S 100 (Evonik Industries) polymer solution, which swells at pH 7. Using the pH-dependent swelling behavior of Eudragit^®^ S 100, phages were released from the dual hydrogel system-coated catheter when urinary pH increased above 7, due to *P. mirabilis* urease [43]. Although the freezing stress during polymer gelation could cause phage inactivation, 4.3 × 10^8^ PFU/mL of phages were released in the first 2 h, suggesting a possible cryoprotective role of PVA.

### 2.4. PVA-SA (Polyvinyl Alcohol-Sodium Alginate) Hydrogel

PVA is extensively used as a drug carrier, owing to its favorable biocompatibility and mechanical properties, but PVA hydrogels lack bioactivity, resulting in weak integration with living tissue as a result of its bio-inert properties (Table 1) [38]. To overcome this drawback, PVA is hybridized with natural hydrogels to integrate advantages of natural and synthetic polymers [38]. For instance, Kaur et al. prepared 10% PVA solution and 3% sodium alginate (SA) solution, mixed at a ratio of 1:2 (PVA: SA) and then crosslinked them using saturated boric acid and 2% CaCl_2_ on cotton gauze. The prepared mixture was frozen at −60 °C and then lyophilized. Subsequently, the freeze-dried gauze was soaked with a phage suspension (1.0 × 10^8^ PFU/mL) and, again, frozen at −60 °C, lyophilized and then stored at 25 °C [26]. Phages were rapidly released from the PVA-SA hydrogel within the first 15 min. However, a significant drop in phage titer (3 log) was observed during the freeze-drying process, probably due to high osmotic pressure created by the increase in osmolarity and osmotic damage [58]. Since PVA may have cryoprotective role [43], the addition of sugars such as sucrose and trehalose may assist in protecting the phages against drying stress [4,59].

### 2.5. HPMC (Hydroxypropyl Methylcellulose) Hydrogel

HPMC is a biodegradable, cellulose-derived natural polymer that can be reversibly crosslinked to form hydrogels upon heating (Table 1) [39]. Kumari et al. produced 3% HPMC hydrogel by dissolving HPMC in warm water, followed by stirring at 100 rpm (rotations per minute) for 15 min until the gel was homogenous in consistency [27,28]. The hydrogel was placed in a vacuum oven at room temperature to remove any entrapped air within the gel and then sterilized by autoclaving at 68.9 kPa for 30 min. The viscosity remained the same after the sterilization procedure. Phage Kpn5 (1 mL of 2.0 × 10^8^ PFU/mL) was mixed with 1 mL of 3% HPMC hydrogel to obtain a phage hydrogel at 1.0 × 10^8^ PFU/mL. The release profile of Kpn5 from the hydrogel was not reported, but in vivo efficacy (see Section 4) demonstrated that 3% HPMC could be an ideal formulation for topical delivery of phages.

### 2.6. Agarose-HAMA (Hyaluronic Acid Methacrylate) Hydrogel

Agarose is a natural polymer that exhibits thermo-reversible gelation behavior similar to HPMC [47], while eliciting robust and inert properties (Table 1) [48]. Agarose hydrogel is widely used as a scaffold in tissue engineering due to excellent biocompatibility and adjustable mechanical properties [47]. However, agarose hydrogel lacks in cell adhesiveness and has poor biodegradability [47]. To overcome this drawback, agarose is often blended with other polymers [48,60]. Bean et al. formulated agarose-hyaluronic acid methacrylate (HAMA) hydrogel system that releases phages in the presence of *Staphylococcus aureus*-specific enzyme hyaluronidase (HAase) [48]. Agarose was suspended in salt magnesium buffer, heated to 95 °C and then cooled down to 50 °C to form 0.78% (*w*/*v*) agarose hydrogel. Then, phage K was added at a ratio of 1:9 (phage:agarose) to give a final phage and agarose concentrations of 1.0 × 10^8^ PFU/mL and 0.7%, respectively. Simultaneously, 2% (*w*/*v*) HAMA solution was mixed with 1% (v/v) PEG diacrylate and 1% (*w*/*v*) Irgacure 2959 was added to the mixture. This HAMA hydrogel was photo-crosslinked with 0.7% (*w*/*v*) agarose hydrogel containing phages under UV light for 60 s. In this study, a HAMA layer served as a barrier, preventing phage release from the agarose layer in the absence of HAase. Although some phages escaped through the pores of the HAMA layer, the majority were released in the presence of HAase, which was accompanied with degradation of cross-linked HAMA. Conversely, the HAMA layer remained intact in the presence of a bacterial strain that did not produce HAase, suggesting that this triggered-release hydrogel system allows semi-automated treatment. This dual system can potentially be applied for other pathogens that produce a unique enzyme.

### 2.7. PNIPAM-co-ALA(N-Isopropylacrylamide-co-Allylamine) Hydrogel

PNIPAM is a thermally responsive polymer that undergoes sol-gel transition (liquid to gel state), where gelation occurs in response to temperature change (Table 1) [46]. At a lower critical solution temperature (32–36 °C), this polymer forms a hydrophobic globule structure as the water is expelled. PNIPAM nanosphere co-polymerized with allylamine provides an interactive surface for phage binding, while regulating the lower critical solution temperature at 37 °C; PNIPAM-co-ALA has a mean diameter of 210 nm at 33 °C, which reduces to 70 nm at 37 °C [46]. Hathaway et al. mixed N-isopropyl acrylamide (10% molar ratio) with allylamine, cross-linker (ethylene-glycol diacrylate, 1% molar ratio) and sodium dodecylsulphate [46]. Subsequently, the solution was freeze-thawed, purged with nitrogen and then heated to 70 °C, followed by addition of sodium persulphate solution (initiator) and was left to polymerize for 4 h. The formulated PNIPAM-co-ALA hydrogel was then grafted to non-woven polypropylene and was soaked with phage K solution (10^9^ PFU/mL) for 4 h at 25 °C. Unlike other phage-delivering hydrogels, the phages are attached to the hydrogel surface and are only released in response to thermal triggers. At 37 °C, PNIPAM-co-ALA nanospheres collapsed and its cargo of phage K was released on a *S. aureus* lawn and formed a zone of clearance, indicating antibacterial activity. Unfortunately, the phage titer in the final product was not investigated in the study.

## 3. Stability of Phages in Hydrogels

Phage stability is an important factor to consider, as this corresponds to the viability of phages over time, which ultimately affects their bactericidal effect. The stability of phages is dependent on many factors, including pH, temperature, formulation composition and light exposure [61]. Phages, being the active pharmaceutical ingredient, must remain biologically stable in the developed formulation over the desired storage time. However, only a few studies have investigated the storage stability of phage hydrogels. In two separate studies, Kumari et al. have shown that *Klebsiella* phage Kpn5 remained stable in a 3% HPMC hydrogel when stored at 37 °C for seven days [27,28]. Similarly, phage LM99 in Alg and Alg-nanoHA hydrogel was biologically stable for seven days (storage temperature unreported) [29]. In another study, *Klebsiella* phage Kpn5, *Pseudomonas* phage PA5 and *Staphylococcus* phage MR10 remained viable in PVA-SA hydrogel when stored at room temperature for 28 days [26]. However, complete inactivation of some phages has been reported in PVA hydrogel, which was thought to be attributed to highly damaging radical species [48].

The field lacks well-controlled long-term storage stability studies of phage hydrogels. In general, neutral polymers are recommended for formulating phages in hydrogels to minimize charge-induced inactivation of phages [62]. Phage capsid has an overall net negative charge and tail a net positive charge at physiological pH. Anionic polymers may unfavorably interact with positively charged tails through electrostatic interaction and block the tail fiber proteins responsible for bacteria binding, compromising phage infectivity. For example, phage formulated in Carbomer (anionic polymer) hydrogel resulted in 99.95% titer reduction within four weeks when stored at 4 °C, whereas those in hydroxyethylcellulose gel (non-ionic) hydrogel remained biologically stable [63]. As only a limited number of hydrogels have been tested for phage stability, in the future, studies on non-ionic hydrogels such as guar gum, agarose, polyethylene oxide, polyvinyl pyrrolidone, polyacrylamide, polycarbophil, poly(hydroxyethyl) methacrylate and hydroxypropyl cellulose could be done.

## 4. Efficacy of Phage-Delivering Hydrogels

### 4.1. Orthopedic Implant-Associated Infection

**Figure 2:**
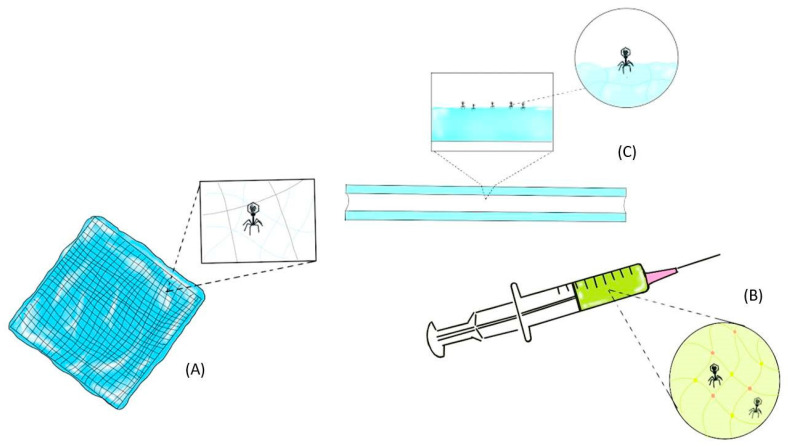
Topical application of phage-delivering hydrogel formulation as a wound dressing (**A**), injectable phage-delivering hydrogel formulation via syringe (**B**) and phage-delivering formulation via a hydrogel-coated catheter (**C**).

Orthopedic implant-associated infections are commonly observed among implanted surgical devices and can cause significant patient morbidity along with financial burden [30]. Virtually all materials used in implantable devices are readily colonized by bacteria and result in biofilm formation with increased resistance to the host immune system and antibiotics [64]. Current treatment options for bone infection are limited to the use of antibiotics and surgical debridement of affected tissue, often followed by implant removal [30]. The use of systematic antibiotics in bone infection is often associated with poor delivery to the site of infection with nephrotoxic and hepatotoxic adverse effects at high dose [65]. To locally deliver MDR bacteria-killing phages, injectable hydrogels have been explored in vivo and in vitro to treat orthopedic implant-associated infections (Figure 2).

Wroe et al. demonstrated that ΦPaer14-encapsulating PEG-4-MAL hydrogel significantly reduced formation of *P. aeruginosa* biofilm (17-fold reduction in colony-forming unit (CFU) counts) as compared with control gels in vitro. Fluorescence staining confirmed that control gels showed a higher load of live bacteria and biofilm-associated proteins, whereas the phage hydrogel-treated group showed higher levels of dead bacteria. Furthermore, phage hydrogel formulated with protease-degradable peptide linkers exhibited rapid killing of planktonic cells in vitro [30]. Antimicrobial efficacy of phage hydrogels can be modulated by utilizing faster degrading peptide sequence, which leads to rapid release of phages for potentially faster elimination of bacteria. In animals with radial segmental defects infected with *P. aeruginosa*, treatment with PEG-4-MAL hydrogel containing a cocktail of four *Pseudomonas* phages exhibited 4.7-fold reduction in bacterial counts (Table 2). Similarly, Johnson et al. applied PEG-4-MAL hydrogel containing both BMP-2 and phages on a mouse radial defect model for 8 weeks, resulting in a significant reduction (>1 log reduction) in the target *P aeruginosa* (PsAer-9) bacteria [41].

In the study conducted by Barros et al., the efficacy of phages LM99 formulated in alginate hydrogel was examined in vitro and ex vivo against MDR *Enterococcus faecalis* isolated from orthopedic implant-associated infections [29]. Treatment with phage hydrogel reduced planktonic cells by 99% and bacterial attachment on hydrogels by 98% after 24 h of incubation. Antibacterial effect was also observed ex vivo with 99.9% reduction in CFU counts after 48 h of treatment with LM99 hydrogel formulation. Additionally, phage-free alginate-nanoHA hydrogel had osteogenic and mineralization response, suggesting that phage hydrogels are a promising multifunctional approach for controlling bacterial infection during implant and bone integration.

Despite promising in vitro and ex vivo data, only limited studies explored the efficacy of phage hydrogels for treatment of orthopedic implant-associated infections in vivo [30,31,41]. Treatment with fosfomycin and/or phage delivered using alginate hydrogel exhibited minimal or no significant antibacterial effect (<1 log reduction in all groups) in soft tissue and bone infection rat models, respectively. Lack of in vitro and in vivo correlation could be due to insufficient delivery and/or release of phages to exert therapeutic effect at the site of infection. The final titer of phage hydrogel was 3.0 × 10^7^ PFU/mL and only 10 μL could fit into the small defect site (i.e., 3.0 × 10^5^ PFU/mL). Hence, a higher initial dose of phages in hydrogels accompanied with controlled release from the gel matrix seems to be essential for in vivo efficacy. Moreover, the use of pig or sheep animal models that best mimic human bones may be more beneficial. In vivo studies often utilized co-administration of both bacteria and phage hydrogels, probably due to the complexity of surgical procedures. A more natural model would be to first inoculate bacteria at the site of interest to create a physical carrier (e.g., biofilms), followed by treatment with phage hydrogels. Further work is required to determine the antimicrobial effect in vivo of phage hydrogels in such chronic orthopedic implant-associated infection.

### 4.2. Catheter-Associated Urinary Tract Infection (CAUTI)

Catheters are commonly used indwelling device in healthcare facilities and are susceptible to biofilm formation, triggering catheter-associated urinary tract infection (CAUTI) [66]. To overcome CAUTI often associated with MDR pathogens, phage-delivering hydrogels are rising as one of the strategies to prevent and eradicate biofilms (Figure 2). Several in vitro studies have demonstrated that phages active against *S. epidermidis, P. aeruginosa, E. coli* and *P. mirabilis* (Curtin/Donlan et al., Fu et al., Carson et al., Lehman/Donlan) can be incorporated into a hydrogel coating on a catheter and significantly reduce biofilm formation [53,54,55,56]. Over 2-log reduction in *P. aeruginosa* biofilm viability was observed upon pre-treatment of the catheter hydrogel with phage M4 [53]. Emergence of phage-resistant biofilm isolate was observed between 24 h and 48 h, but the use of a five-phage cocktail significantly suppressed this phenomenon. A study by Curtin and Donlan has shown that phage 456 incorporating a hydrogel-coated catheter can reduce *S. epidermidis* biofilm formation over a 24 h exposure with log CFU/cm^2^ reduction of 4.5 (Table 3). Interestingly, supplemental divalent cations along with phage 456 fostered further reduction in *S. epidermidis* cell attachment with log CFU/cm^2^ reduction of 2.3 as compared with phage-free controls [55]. Divalent cations such as Ca^2+^ or Mg ^2+^ are known for promoting growth or enhancing the antibacterial activity of phage [55,57,67]. Although phage-dependent, divalent cations can aid in phage attachment to host bacteria [49]. In gel formulations, divalent cations are used as crosslinking agents, yet these cations may not be freely available for interaction with phages. Hence, supplemental addition of cations after hydrogel formulation could be considered to enhance the antibacterial activity of phages.

Multispecies biofilm can form on a catheter [68,69] and act as a stable reservoir of various pathogens that are difficult to eliminate. Lehman and Donlan [56] used a phage cocktail comprising six *P. aeruginosa* phages (1.0 × 10^9^ PFU/mL each) and/or four *P. mirabilis* phages (3.0 × 10^8^ PFU/mL each) (Table 3) on a hydrogel-coated catheter to target single and multispecies biofilms. Treatment with *Pseudomonas* phage cocktail reduced single-species biofilm levels by 2.5 log after 24 h as compared with buffer-treated control, followed by regrowth at 48 h (1.5 log reduction). Antibiofilm activity of the cocktail was more pronounced against two-species biofilm with 3 log and 4 log reductions at 24 h and 48 h, respectively. Similarly, treatment with a *Proteus* phage cocktail reduced *P. mirabilis* populations in both single and multispecies biofilms. Interestingly, increased pH due to *P. mirabilis* urease activity caused elimination of *P. aeruginosa* by 72 h regardless of phage treatment, suggesting that observed efficacy against multispecies biofilms could be due to interplay of multiple factors. Increased pH caused by bacterial urease leads to supersaturation and precipitation of struvite and apatite crystals, forming crystalline biofilms that can block urinary catheters. A simple PVA-based phage hydrogel further formulated with pH-responsive polymer delayed the catheter blockage time (26 h) caused by *P. mirabilis* biofilms as compared with non-treated control (13 h) [43]. The majority of these studies used 24 h old biofilms, which are considered quite young. The efficacy against mature biofilms (≥48 h) with more complex and persistent extracellular matrix should be investigated in the future. Furthermore, adjunctive use of other anti-biofilm agents, such as antibiotics [70], nitric oxide [71,72] and amino acids [73,74], could be considered for a synergistic antibacterial effect for treating chronic infections.

### 4.3. Trauma-Associated Skin and Soft Tissue Infection

Wounds from burn injuries provide a favorable environment for the growth of bacteria [27]. Burn injuries contribute to the fourth leading devastating form of trauma worldwide and may lead to death if untreated [26]. Topical application of antimicrobial agents is preferred over systemic antibiotics due to high bioavailability at the site of infection [28]. Hydrogels are commonly used in wound care products as they promote wound healing, maintain a hydrated environment essential for self-healing and clear debridement while absorbing the exudates [75]. Phages have been incorporated in hydrogels to treat bacterial wound infections (see Figure 2; their topical application has been extensively reviewed by Chang et al. [62]).

Phage K formulated in HAMA/agarose hydrogel system exhibited a clear zone of inhibition on *S. aureus* bacterial lawn [48]. Furthermore, temperature-sensitive PNIPAM-co-ALA hydrogel attached to non-woven polypropylene could deliver infective phage K and form a bacterial zone of clearance, indicating in vitro efficacy of the formulation (Table 4) [46]. In another study, treatment with PVA-SA hydrogel-based membrane containing phages MR10, Kpn5 and PA5 (all at a reported MOI of 10) resulted in 6 log reduction in *S. aureus*, 6.37 log reduction in *Klebsiella pneumoniae* and 4.6 log reduction in *P. aeruginosa* biomass, respectively, after 6 h in vitro (Table 4) [26]. Treatment with MR10 hydrogel resulted in fast wound healing in *S. aureus* burn wound infection model in mice as compared with non-treated control, particularly when minocycline was used in combination. On day 14, those receiving dual-agent treatment showed complete regeneration of skin layers, sweat glands and hair follicles similar to normal mouse skin. In addition to rapid wound healing, phage hydrogels significantly reduced mortality in mice burn wound infection model [28]. A study by Kumari et al. demonstrated that treatment with phage Kpn5 in HPMC hydrogel (MOI of 200) increased the survival rate of *Klebisella*-infected animals as compared with other antimicrobials, including silver nitrate and gentamicin (Table 4). All mice in the phage hydrogel treated group survived, while 87% survived in the non-treated group on day 1. On day 7, the phage-treated group showed the highest level of protection (63%) as compared with the untreated group (0%). The survival rate may be further increased by the combined use of phages and antibiotics.

Despite demonstrating prominent antibacterial activity, phage-delivering hydrogels were limited to targeting only one species in the context of combating wound infections. As wound infections are often polymicrobial [76], future studies should include phage cocktail consisting of prominent bacterial isolates in wound infections, such as *P. aeruginosa*, methicillin-resistant *S. aureus* and *K. pneumoniae*. Subsequent multispecies wound infection model should be developed and utilized to better reflect the clinical setting. Furthermore, formulation of hydrogels containing both phage and antibiotic should be considered to maximize bactericidal activity, while minimizing phage- and antibiotic-resistant isolates. Overall, most of the phage-delivering hydrogels demonstrated significant efficacy against MDR bacterial infection in vivo and in vitro. Aligned with efficacy, safety of phage-delivering hydrogels needs to be considered to further progress into human clinical trials.

## 5. Safety of Phage-Delivering Hydrogels

For any drug to undergo clinical trials, its safety profile must be evaluated. Phages are generally considered safe provided the preparation is sufficiently purified with endotoxins and host cell proteins removed [77]. Safety profile of phage hydrogels has been investigated by assessing cytocompatibility [26,29], hemocompatibility [26], cell cytotoxicity [26,30] and the presence of inflammatory response on the murine and rabbit skin [26,27,28,29,30]. However, the safety profile of phage-free hydrogels has been more extensively studied as part of the formulation optimization step prior to incorporation of phages [26,27,28,29]. In general, various hydrogels (e.g., PVA-SA [26], DNA [78], poly(2-dimethylamino-ethylmethacrylate) [78], PVA-dextran [79]) had good hemocompatibility and a non-hemolytic nature. PVA-SA hydrogel was compatible with skin epithelial and macrophage cells with over 95% cell viability after 24 h and did not cause any irritation (i.e., no rash or edema formation) on murine skin, suggesting the safety of the optimized formulation for topical application [26]. Similarly, studies by Kumari et al. have shown that 3% HPMC hydrogel was safe with no development of rash, inflammation, swelling, scaling and abnormal tissue growth on mice skin for up to seven days [27,28]. However, the safety data of both PVA-SA and HPMC hydrogels were based on the absence of phages. Therefore, after incorporating phages, these hydrogels could potentially induce inflammatory responses and compromise cell viability in vivo due to the presence of contaminants such as bacterial genes, proteins and endotoxins in the phage lysate.

For the development of commercial products, the purification of phage preparations is a critical step if the gel formulation is delivered via parenteral or topical routes. For example, Wroe et al. developed ΦPaer14-encapsulating PEG-4-MAL hydrogels for the treatment of orthopedic-implant associated infections. Phage purification steps consisted of filtration, followed by anion-exchange chromatography and then removal of residual endotoxin using EndoTrap column (Hyglos) [30]. Although the endotoxin levels in the final product was not reported, given the current endotoxin acceptance criteria for intravenous administration is 5 EU/kg/h [80], and a similar standard could be taken for injectables. In fact, ΦPaer14-encapsulating PEG-4-MAL hydrogel was cytocompatible with human mesenchymal stromal cells in vitro and the metabolic activity of these cells remained unchanged for 72 h. Furthermore, the formulation was well tolerated at bone defect sites in vivo regardless of the presence of phages, with no differences in the surrounding tissue for up to four weeks. In another study, the safety profile of phage-loaded alginate-nanohydroxyapatite hydrogel formulated for the prevention of orthopedic implant-associated infections was assessed [29]. Although the phage purification method was not disclosed in the study, the formulation was cytocompatible with human osteoblastic cells, and no complication was observed in animals with subcutaneous implantation of phage hydrogels. Moreover, no significant inflammatory response nor cellular exudate was observed at the implantation site. At 6 weeks after the implantation, no differences in the immune response, tissue infiltration and recruited immune cell population could be detected in all animals. These studies provide preliminary evidence that phage hydrogel formulations are safe for topical application on skin as well as subcutaneous injection. In addition, hydrogels offer protection against immune recognition and subsequent clearance of phages from the site of infection [33]. The effect of long-term exposure is yet to be elucidated.

## 6. Future Perspective

Phage hydrogels are gaining attention in medical fields in recent years, revealing promising potential for clinical use. Various types of polymers and functional hydrogel formulations have been explored as potential vehicle for delivering phages to target MDR bacterial infections. As discussed in Section 2, hydrogels can be formulated using various physical and chemical methods, and these manufacturing processes may impact phage stability. To enhance the likelihood of phage stabilization in hydrogels, phage incorporation should be done after formulation of hydrogels whenever feasible. Regardless of the phage type used, the physical properties of hydrogel will likely remain the same as the mass fraction of the phages is minimal in the formulation. Depending on the composition of hydrogel and manufacture process, physical properties such as pore size distribution and porosity can be controlled, which ultimately affects the amount of phage released from the hydrogels. The release profile of phages, in turn, is expected to correlate with the efficacy of phage therapy. While the biocompatibility of hydrogels with human cells is extensively explored, phage hydrogels have been understudied. However, phage hydrogels are anticipated to be compatible with human cells if the phage preparation is sufficiently purified and free of bacterial genes and proteins. Storage stability of phages in hydrogel has been underexplored and each phage may exhibit a different viability profile. As liquid formulation of phages often requires refrigeration, storage at 4 °C could also be considered for phage hydrogels. Phage viability could vary in different hydrogels depending on the nature or due to specific components of hydrogels. Therefore, long-term stability and safety data with a wide range of hydrogel types could be investigated in future studies to provide comprehensive characterization of phage hydrogels. Based on the early literature, which dates back to the 1960s, individual phages may exhibit a different stability profile regardless of their morphology or family they belong to [23]. Nonetheless, further research on phage formulation will be beneficial in providing a basic formulation strategy, which can then be optimized for the target phage of interest. Finally, phage hydrogels could expand their role to delivery of phages to target bacterial infections associated with ophthalmic, dental and auditory systems.

## 7. Conclusions

Phage therapy is gaining attention as a viable solution for combating MDR bacteria. To deliver phages, hydrogels have been investigated considering their low toxicity, good biocompatibility, resemblance to the living cell environment and modifiable physicochemical properties. Hydrogels as a phage carrier have shown promising effects regarding targeting orthopedic implants, catheter and trauma-associated infections in the available literature. However, further research is urgently needed to investigate phage-delivering hydrogels, particularly with respect to their stability and safety in order to be utilized in clinical settings. Additionally, future studies should test a wider variety of hydrogels for phage delivery.

## Figures and Tables

**Table 1:** Application and preparation of hydrogels used for delivering phages.

Application	Polymer	Preparation Method	Characteristic	Target Bacteria	Phages	References
Wound dressing	PVA-SA	Ion crosslinking (physical crosslinking)	Good mechanical property Excellent hemostatic property Good biodegradability	*S. aureus* *P. aeruginosa* *K. pneumoniae*	MR10 PA5 Kpn5	[26,38]
Wound dressing	HPMC	Thermal gelation (physical crosslinking)	Thermo-reversible gelation Good biodegradability	*K. pneumoniae*	Kpn5	[27,28,39]
Injectable	PEG-4-MAL	Michael-type addition (chemical crosslinking)	Rapid gelation Robust and bioactive network Good biodegradability	*P. aeruginosa*	ΦPaer4 ΦPaer14 ΦPaer22 ΦW2005A	[30,40,41]
Wound dressing Injectable	Alginate	Ion crosslinking (physical crosslinking)	Resist low pH Good biodegradability	*S. aureus* *E. faecalis*	Genetically modified phage vB_EfaS_LM99	[29,31,37]
Coating urinary catheter	PVA- Eudragit^®^ S 100	Freezing and thawing (physical crosslinking)	Good mechanical property Poor biodegradability Low cell adhesion	*P. mirabilis*	Phage isolated from sewage	[42,43]
Coating urinary catheter	PEG- polyurethane	Bulk polymerization (chemical crosslinking)	Thermo-responsive Anti-biofouling Poor biodegradability	*S. epidermidis*	Phage 456	[44,45]
				*E. coli*	Coli-proteus phage	
				*P. aeruginosa*	ΦPaer4, ΦPaer14, M4, 109, ΦE2005-A, ΦE2005-C	
				*P. mirabilis*	ΦPmir1, ΦPmir32, ΦPmir34, ΦPmir37, T4	
Wound dressing	PNIPAM-co- ALA	Thermal gelation (physical crosslinking)	Thermo-reversible gelation Poor biodegradability	*S. aureus*	Phage K	[46]
Wound dressing	Agarose-HAMA hydrogel	Thermal gelation (physical crosslinking)	Thermo-reversible gelation Poor biodegradability Low cell adhesion	*S. aureus*	Phage K	[47,48]

Note: PVA-SA, polyvinyl alcohol-sodium alginate; HPMC, hydroxypropyl methylcellulose; PEG-4-MAL, polyethylene glycol-4-maleimide; PVA, polyvinyl alcohol; PEG-polyurethane, polyethylene glycol-polyurethane; PNIPAM-co-ALA, N-isopropylacrylamide-co-allylamine; HAMA, hyaluronic acid methacrylate.

**Table 2:** Phage-delivering hydrogels for the prevention of orthopedic implant-associated bone infections.

Hydrogel	Target Bacteria	Phages	Study	Key Findings	References
Alginate hydrogel, alginate nanohydroxyapatite hydrogel	*E. faecalis*	vB_EfaS_LM99	In vitro and in vivo (rabbit)	Inhibited the attachment and colonization of MDR *E. faecalis* in femoral tissues. Inhibited growth (99.9%) of planktonic cells after 48 h.	[29]
PEG-4-MAL hydrogel	*P. aeruginosa*	ΦPaer4, ΦPaer14, ΦPaer22, ΦW2005A	In vitro and in vivo (mouse)	Reduced *P. aeruginosa* growth (4.7-fold) at the infection site after 7 days of implantation in mice	[30]
Alginate hydrogel	*S. aureus*	Genetically modified phage	In vitro and in vivo (rat)	No significant reduction in bone infection.	[31]

Note: PEG-4-MAL, polyethylene glycol-4-maleimide; MDR, multidrug-resistance.

**Table 3:** Summary of phage-delivering hydrogels in catheter-associated urinary tract infection.

Therapeutic Use	Hydrogel	Target Bacteria	Phages	Study Design	Findings	References
Preventing catheter-associated UTI	PEG-polyurethane hydrogel	*P. aeruginosa*	*Pseudomonas* phage cocktail: ΦPaer4, ΦPaer14, M4, 109, ΦE2005-A and ΦE2005-C	In vitro	Reduced formation of multi-species biofilm comprising *P. aeruginosa* (4 log) and *P*. *mirabilis* (2 log) in an artificial urine medium with 72 and 96 h exposure	[56]
		*P. mirabilis*	*Proteus* phage cocktail: ΦPmir1, ΦPmir32, ΦPmir34, ΦPmir37			
		*S. epidermidis*	Phage 456	In vitro	Reduced biofilm formation over a 24 h exposure with a log reduction of 4.47	[55]
		*P. mirabilis* *E. coli*	T4 Coli-proteus phage	In vitro	Reduced biofilm formation by approximately 90%	[54]
		*P. aeruginosa*	Phage M4	In vitro	Reduced biofilm formation and bacterial attachment to catheter. Phage cocktails on hydrogel-coated catheter reduction of 99.9% on biofilms composed of 11 variants after 48 h.	[53]
Preventing encrustation of catheter lumen and catheter-associated UTI	PVA- Eudragit^®^ S 100 hydrogel	*P. mirabilis*	Phage isolated from crude sewage	In vitro	Phage-delivering hydrogel reduced the *P. mirabilis* biofilm formation by 6 log reduction	[43]

Note: PEG-polyurethane, polyethylene glycol-polyurethane; PVA, polyvinyl alcohol; UTI, urinary tract infection.

**Table 4:** Summary of phage-delivering hydrogels in trauma-associated skin and soft tissue infection.

Therapeutic Use	Hydrogel	Target Bacteria	Phages	Study Design	Findings	References
Treating wound associated with burn injury	HPMC hydrogel	*K. pneumoniae*	Kpn5	In vivo (mice)	The highest survival rate compared to silver nitrate and gentamicin after 7 days	[28]
	PVA-SA hydrogel	*S. aureus* *P. aeruginosa* *K. pneumoniae*	MR10 PA5 Kpn5	In vitro and in vivo (mice)	Reduced resistant burn wound infection significantly (>1 log reduction) and exhibited reduction in inflammation with wound contraction.	[26]
Treating skin infection	Agarose-HAMA hydrogel	*S. aureus*	Phage K	In vitro	Triggered release of phage K under the presence of hyaluronidase, degrading the HAMA layer, and thus, inhibiting bacterial growth	[48]
Treating skin and soft tissue infection	PNIPAM-co-ALA hydrogel	*S. aureus*	Phage K	In vitro	PNIPAM-co-ALA nanogels attached to phage K exhibited thermally triggered bacterial lysis of *S. aureus* at 37 °C.	[46]
Treating orthopedic implant-associated soft tissue infection	Alginate hydrogel	*S. aureus*	Genetically modified phage	In vitro and in vivo (rat)	Reduced soft tissue infection significantly (>0.5 log reduction)	[31]

Note: PVA-SA, polyvinyl alcohol-sodium alginate; HPMC, hydroxypropyl methylcellulose; PNIPAM-co-ALA, N-isopropylacrylamide-co-allylamine; HAMA, hyaluronic acid methacrylate.

## Data Availability

Not applicable.

## Abbreviations

Alg, alginate solution; CAUTI, catheter-associated urinary tract infection; CFU, colony-forming unit; HAase, hyaluronidase; HAMA, hyaluronic acid methacrylate; LM99, vB_EfaS_LM99;MDR, multidrug-resistant; PEG, polyethylene glycol; PEG-4-MAL, polyethylene glycol-4-maleimide; PFU, plaque forming units; phage, bacteriophage; PNIPAM-co-ALA, N-isopropylacrylamide-co-allylamine; PVA, polyvinyl alcohol; PVA-SA, polyvinyl alcohol-sodium alginate; Rpm, rotations per minute; SA, sodium alginate; VPM, GCRDVPMSMRGGDRCG.
